# Supplementary figures and images for: Strip1 regulates retinal ganglion cell survival by suppressing Jun-mediated apoptosis to promote retinal neural circuit formation
Source: eLife. 2022 Mar 22;11:e74650. doi: 10.7554/eLife.74650 (PMC8940179; doi:10.7554/eLife.74650)

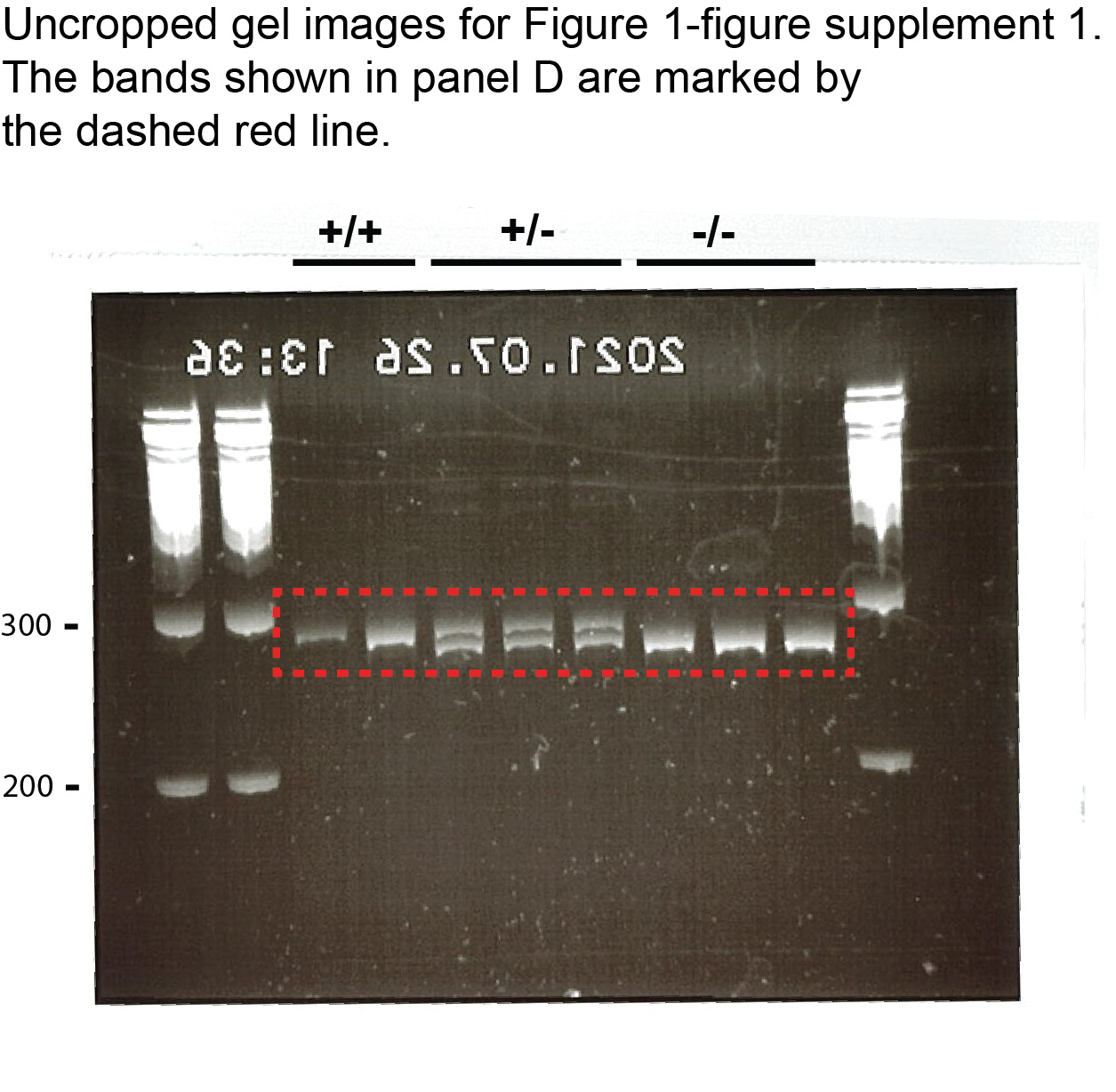

Supplement: Figure 1—figure supplement 1—source data 1. [file elife-74650-fig1-figsupp1-data1.zip › Data for Figure 1-figure supplement 1D/Data for Figure 1ΓÇôfigure supplement 1D (uncropped gel image).jpg]

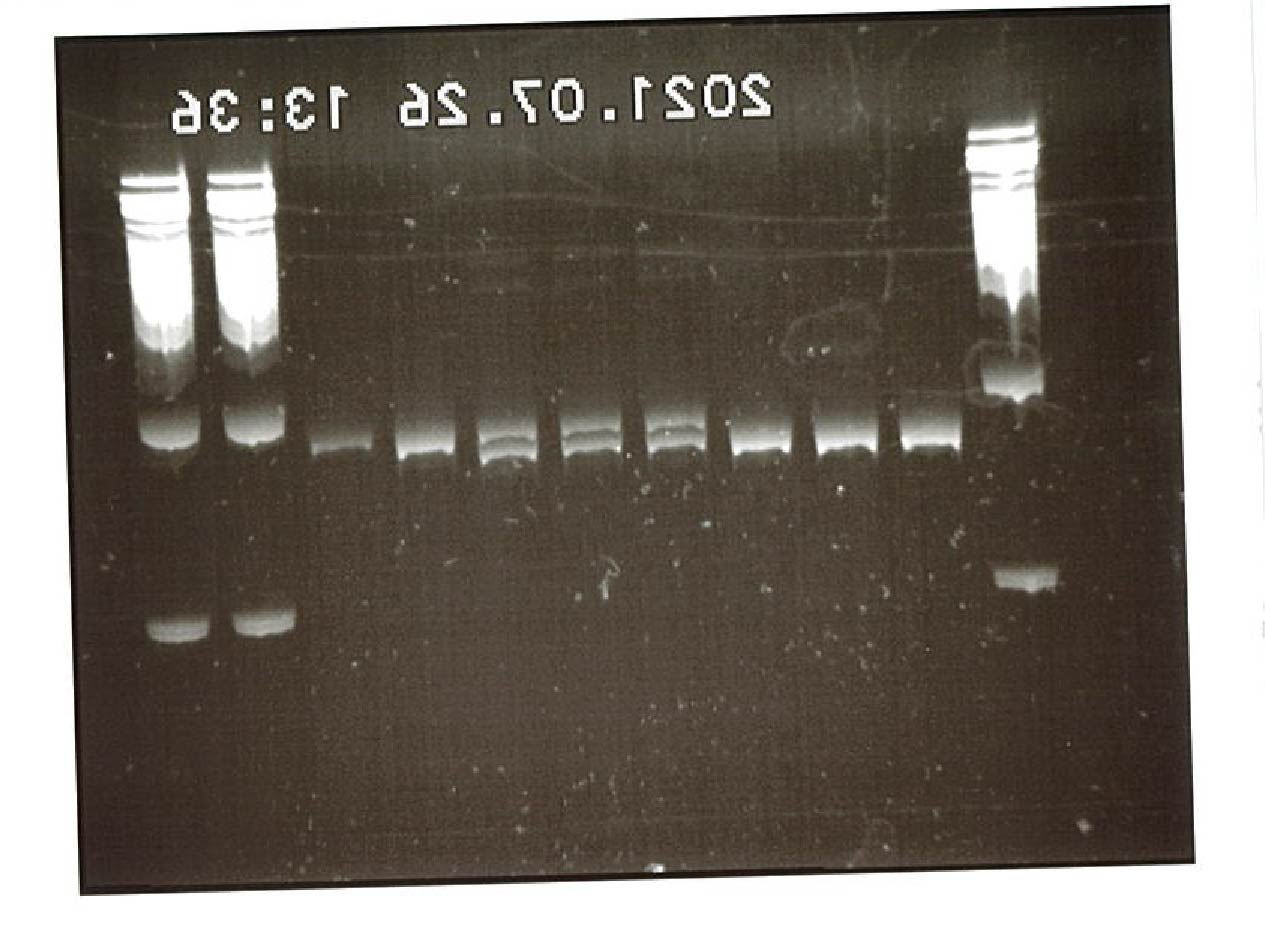

Supplement: Figure 1—figure supplement 1—source data 1. [file elife-74650-fig1-figsupp1-data1.zip › Data for Figure 1-figure supplement 1D/Data for Figure 1-figure supplement 1D (original gel).jpg]

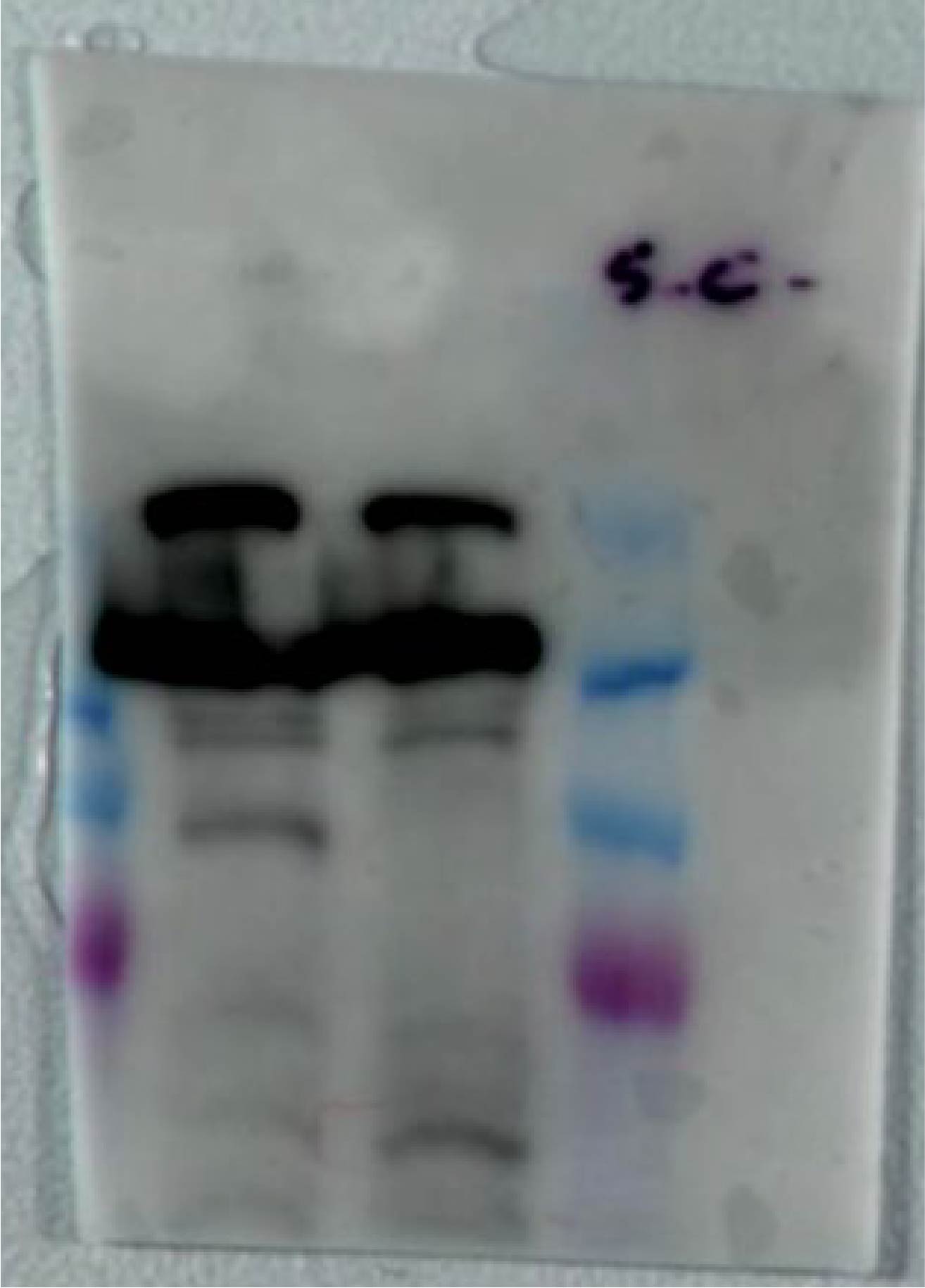

Supplement: Figure 1—figure supplement 1—source data 2. [file elife-74650-fig1-figsupp1-data2.zip › Data for Figure 1-figure supplement 1G/Data for Figure 1-figure supplement 1G (original blot strip1).jpg]

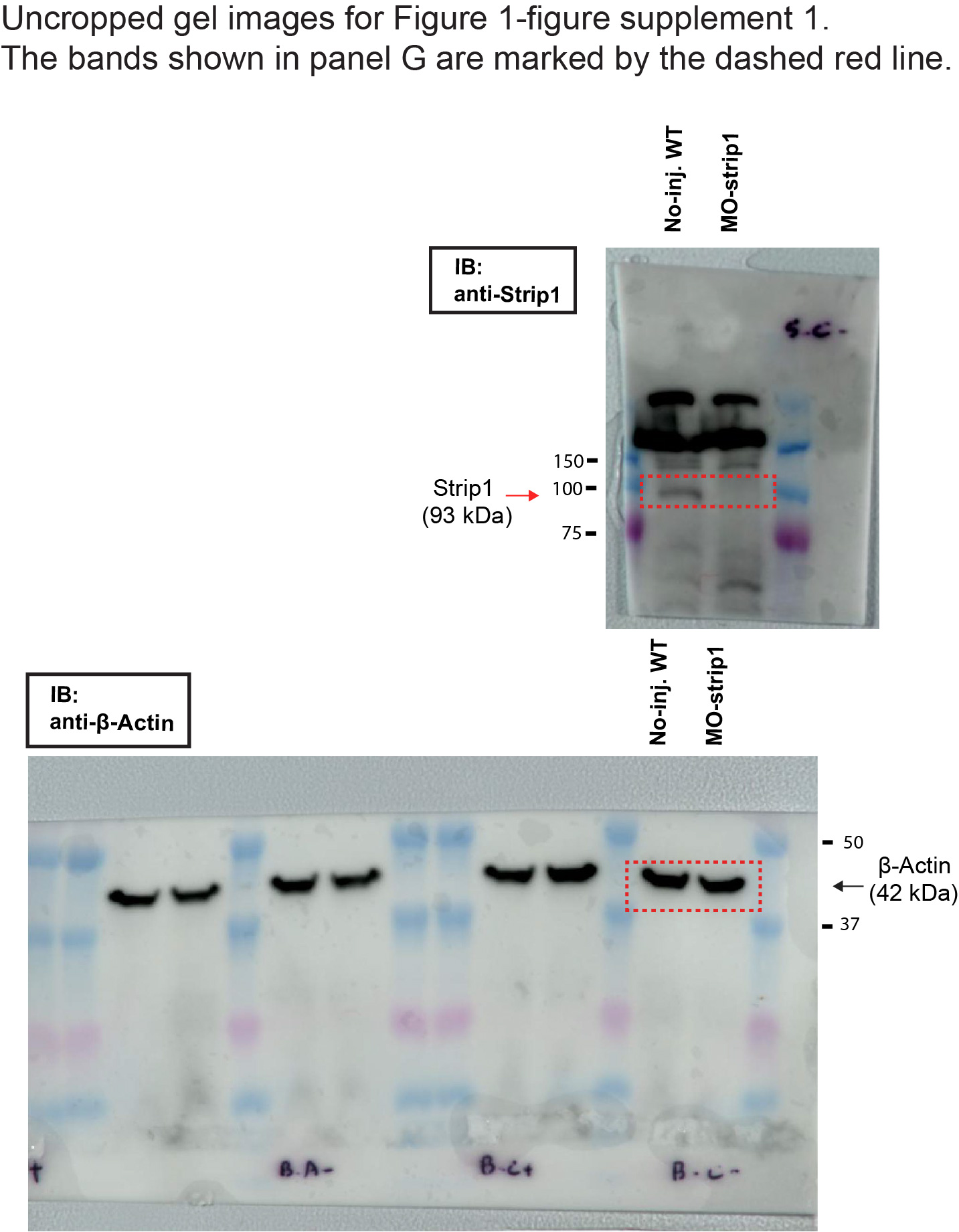

Supplement: Figure 1—figure supplement 1—source data 2. [file elife-74650-fig1-figsupp1-data2.zip › Data for Figure 1-figure supplement 1G/Data for Figure 1ΓÇôfigure supplement 1G (uncropped blot image).jpg]

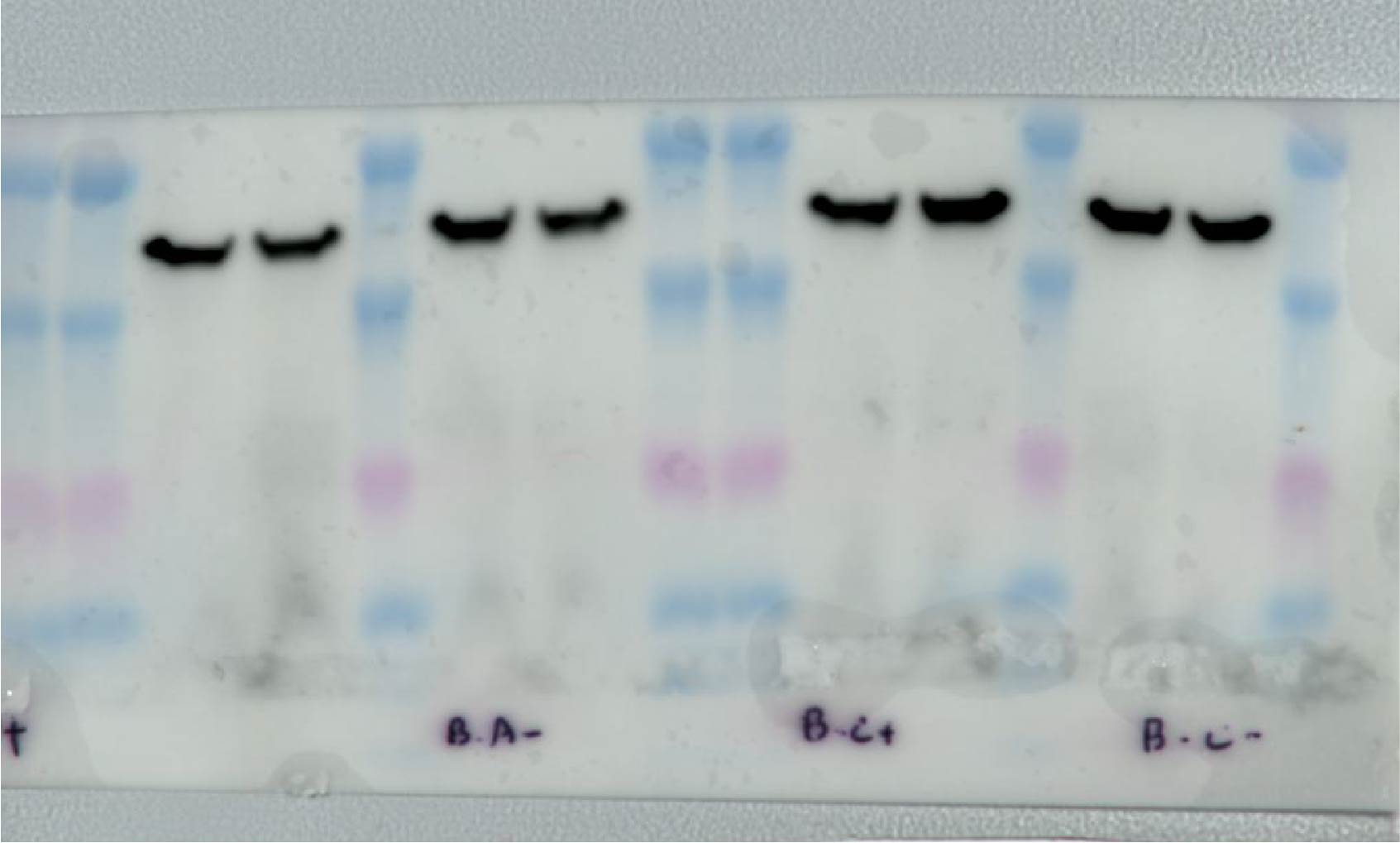

Supplement: Figure 1—figure supplement 1—source data 2. [file elife-74650-fig1-figsupp1-data2.zip › Data for Figure 1-figure supplement 1G/Data for Figure 1-figure supplement 1G (original blot beta-actin).jpg]

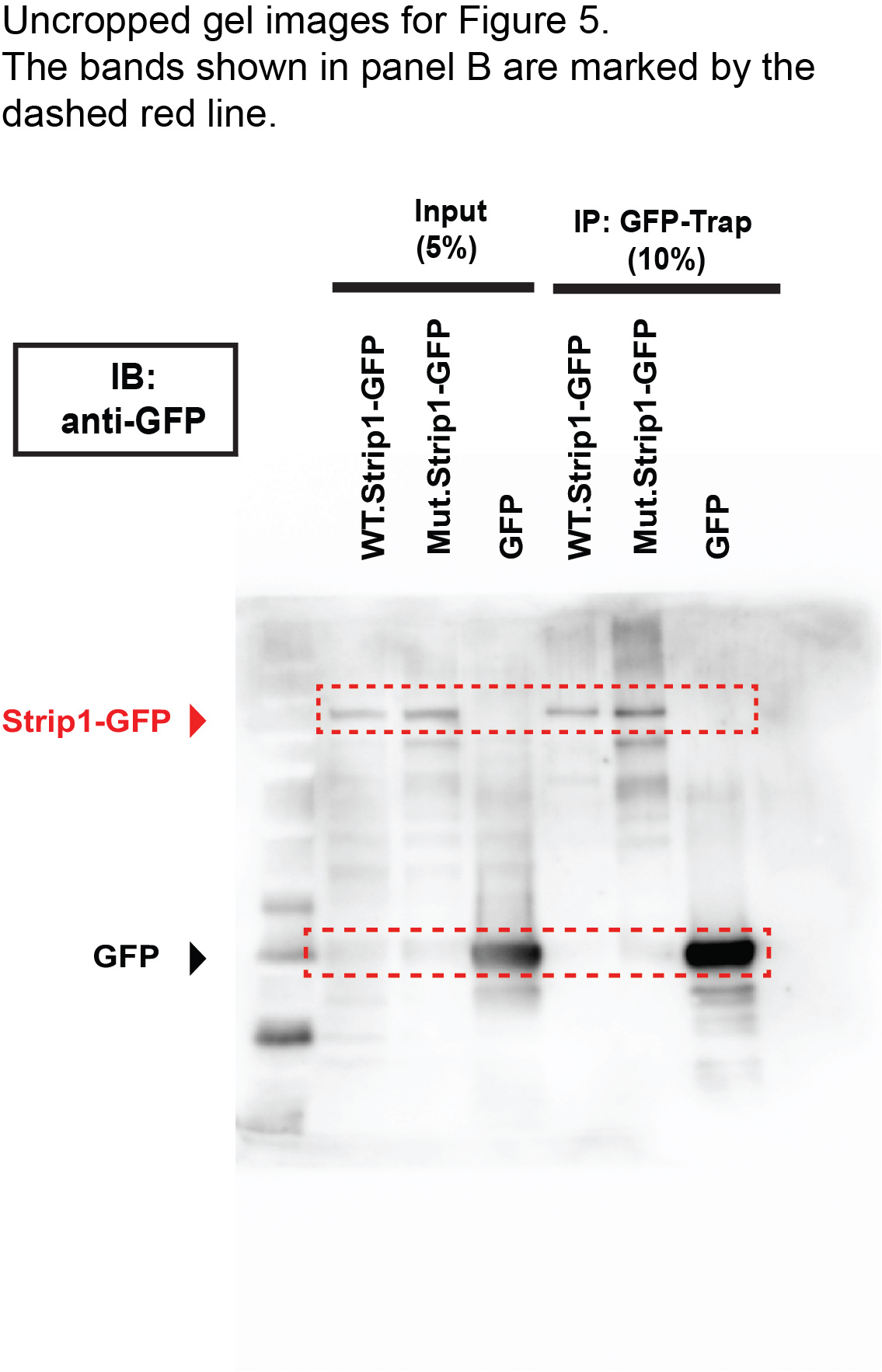

Supplement: Figure 5—source data 1. [file elife-74650-fig5-data1.zip › Data for Figure 5B/Data for Figure 5B (uncropped blot image).jpg]

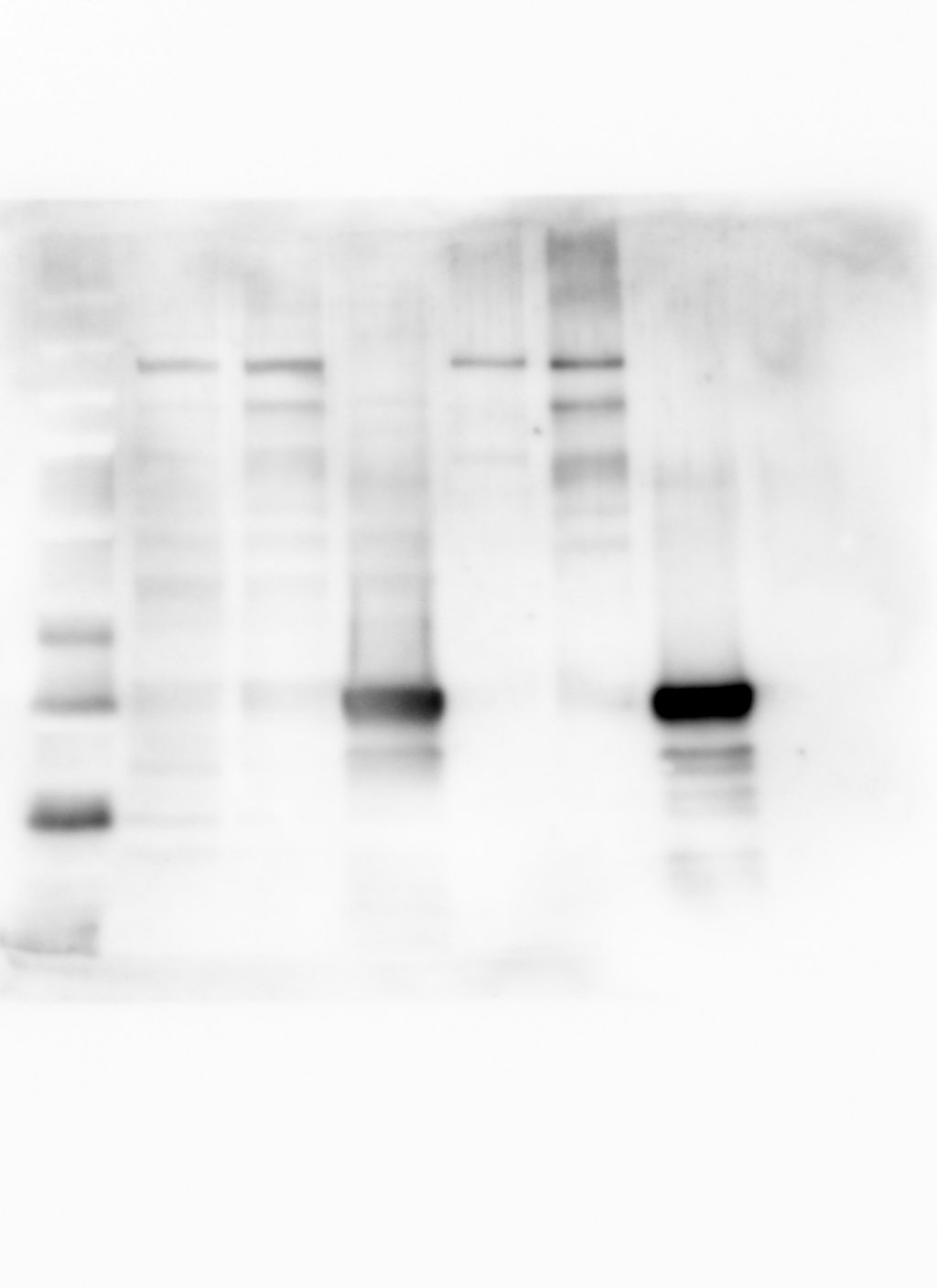

Supplement: Figure 5—source data 1. [file elife-74650-fig5-data1.zip › Data for Figure 5B/Data for Figure 5B (original blot).png]

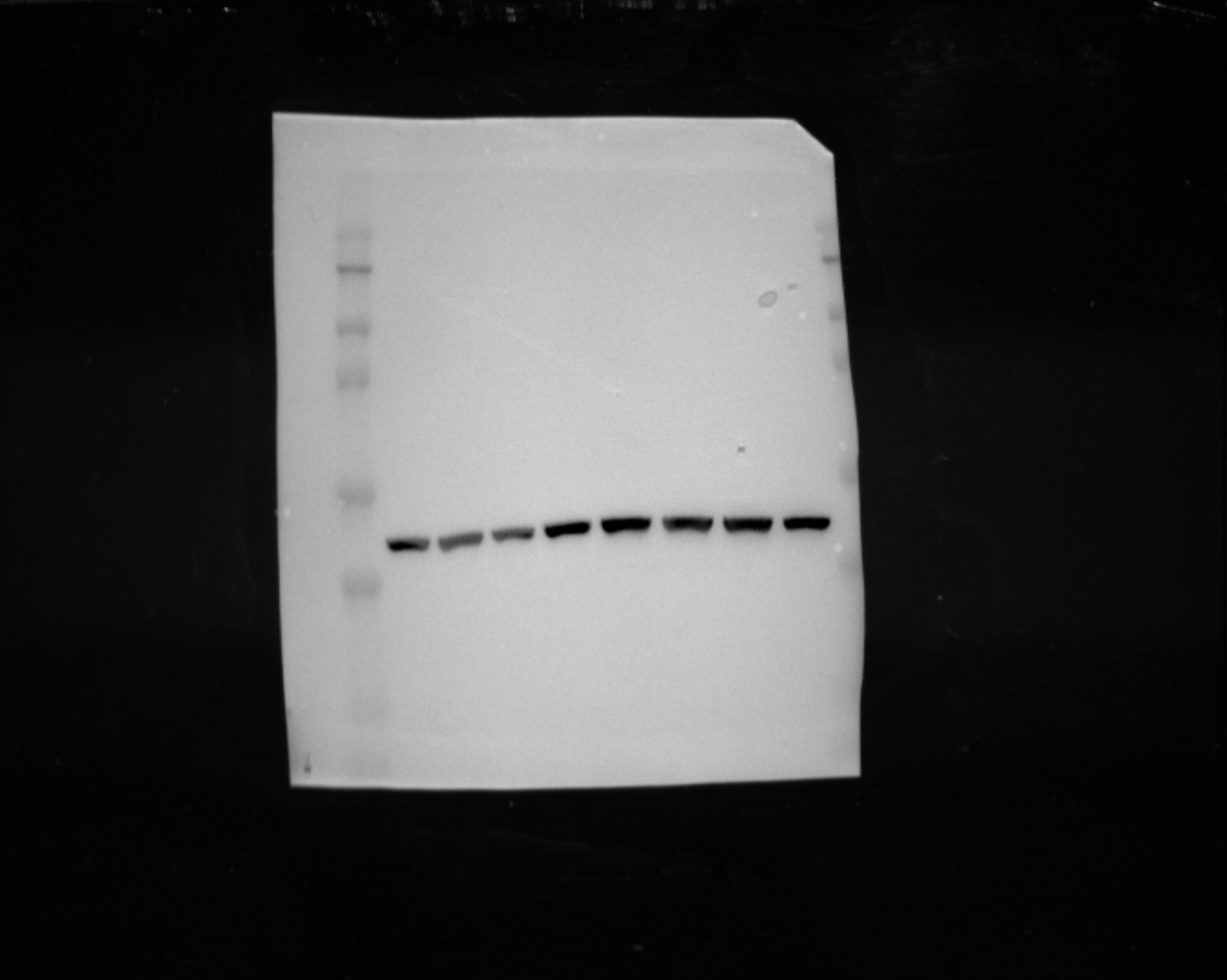

Supplement: Figure 5—figure supplement 2—source data 1. [file elife-74650-fig5-figsupp2-data1.zip › Data for Figure 5-figure supplement 2A/Figure 5-figure supplement 2A (original blot_Beta-actin).tif]

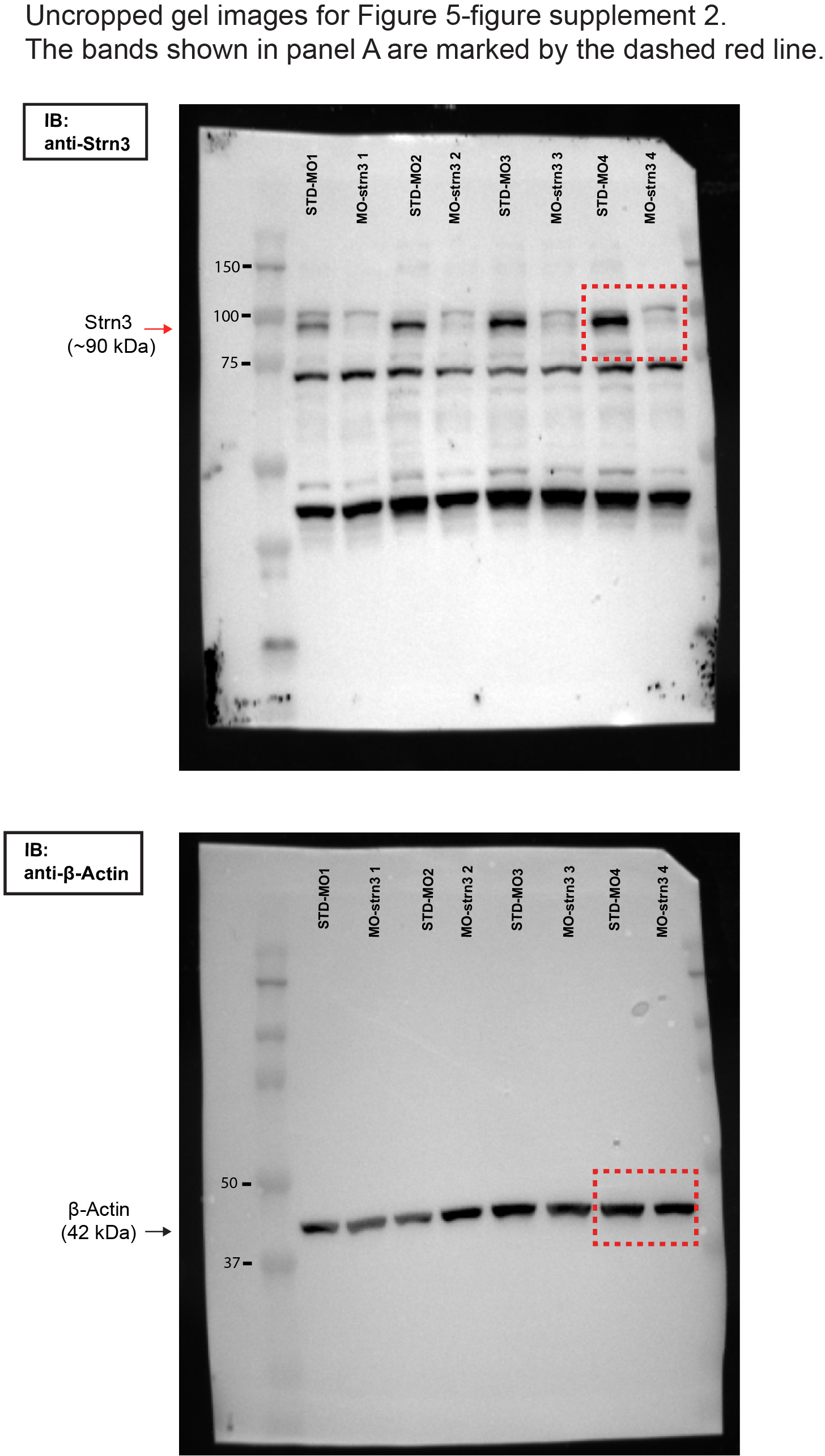

Supplement: Figure 5—figure supplement 2—source data 1. [file elife-74650-fig5-figsupp2-data1.zip › Data for Figure 5-figure supplement 2A/Figure 5-figure supplement 2A(uncropped blot images).jpg]

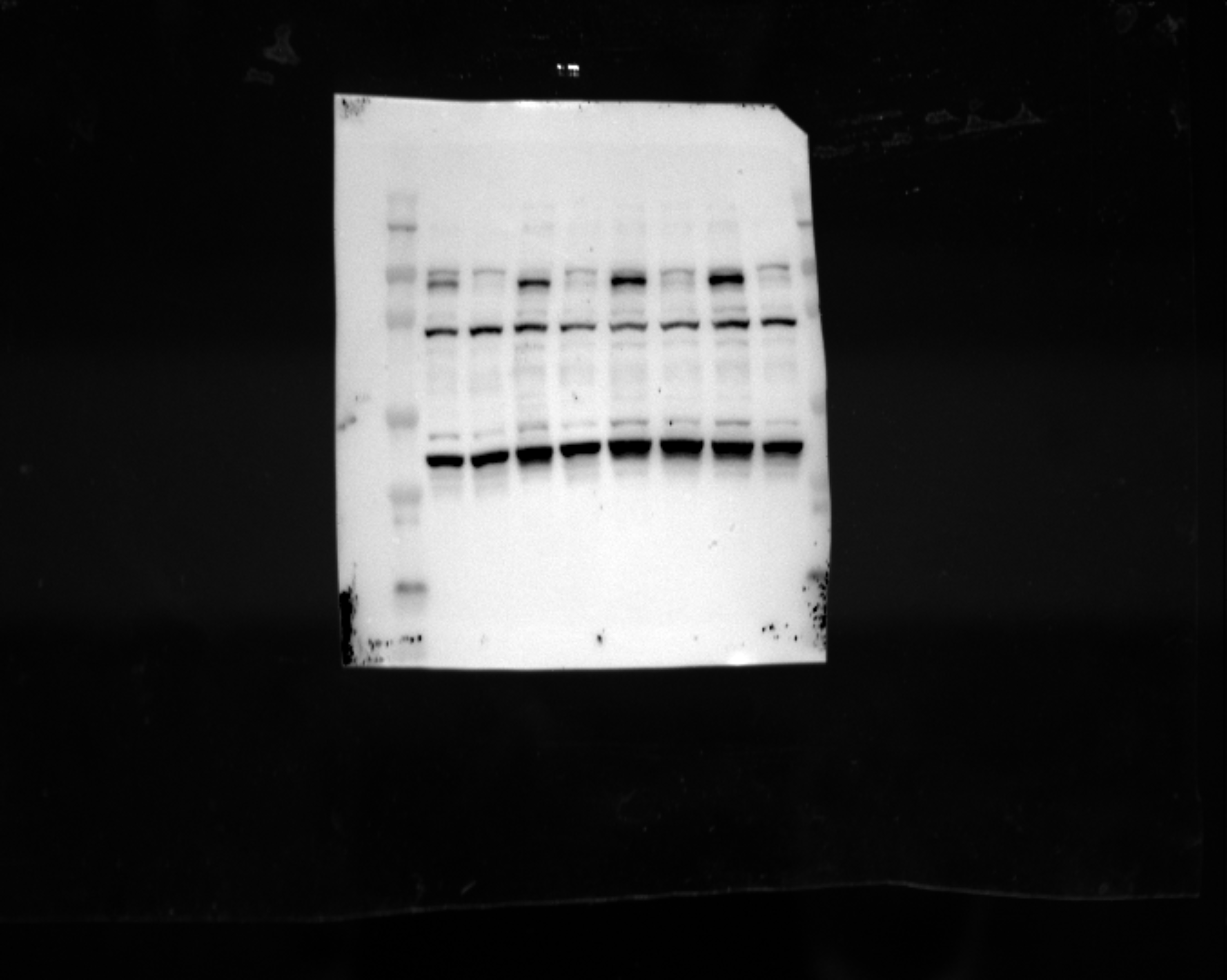

Supplement: Figure 5—figure supplement 2—source data 1. [file elife-74650-fig5-figsupp2-data1.zip › Data for Figure 5-figure supplement 2A/Figure 5-figure supplement 2A (original blot_Strn3).tif]
